# CNstream: A method for the identification and genotyping of copy number polymorphisms using Illumina microarrays

**DOI:** 10.1186/1471-2105-11-264

**Published:** 2010-05-19

**Authors:** Arnald Alonso, Antonio Julià, Raül Tortosa, Cristina Canaleta, Juan D Cañete, Javier Ballina, Alejandro Balsa, Jesús Tornero, Sara Marsal

**Affiliations:** 1 Grup de Recerca de Reumatologia, Institut de Recerca de l'Hospital Universitari Vall d'Hebrón (UAB), Barcelona, Spain; 2 Hospital Clinic i Provincial de Barcelona, Barcelona, Spain; 3 Hospital Universitario Central de Asturias, Oviedo, Asturias, Spain; 4 Hospital Universitario La Paz, Madrid, Spain; 5 Hospital Universitario de Guadalajara, Castilla-La Mancha, Spain

## Abstract

**Background:**

Understanding the genetic basis of disease risk in depth requires an exhaustive knowledge of the types of genetic variation. Very recently, Copy Number Variants (CNVs) have received much attention because of their potential implication in common disease susceptibility. Copy Number Polymorphisms (CNPs) are of interest as they segregate at an appreciable frequency in the general population (i.e. > 1%) and are potentially implicated in the genetic basis of common diseases.

**Results:**

This paper concerns CNstream, a method for whole-genome CNV discovery and genotyping, using Illumina Beadchip arrays. Compared with other methods, a high level of accuracy was achieved by analyzing the measures of each intensity channel separately and combining information from multiple samples. The CNstream method uses heuristics and parametrical statistics to assign a confidence score to each sample at each probe; the sensitivity of the analysis is increased by jointly calling the copy number state over a set of nearby and consecutive probes. The present method has been tested on a real dataset of 575 samples genotyped using Illumina HumanHap 300 Beadchip, and demonstrates a high correlation with the Database of Genomic Variants (DGV). The same set of samples was analyzed with PennCNV, one of the most frequently used copy number inference methods for Illumina platforms. CNstream was able to identify CNP loci that are not detected by PennCNV and it increased the sensitivity over multiple other loci in the genome.

**Conclusions:**

CNstream is a useful method for the identification and characterization of CNPs using Illumina genotyping microarrays. Compared to the PennCNV method, it has greater sensitivity over multiple CNP loci and allows more powerful statistical analysis in these regions. Therefore, CNstream is a robust CNP analysis tool of use to researchers performing genome-wide association studies (GWAS) on Illumina platforms and aiming to identify CNVs associated with the variables of interest. CNstream has been implemented as an R statistical software package that can work directly from raw intensity files generated from Illumina GWAS projects. The method is available at http://www.urr.cat/cnv/cnstream.html.

## Background

Over the last three years, Single Nucleotide Polymorphism (SNP)-based GWAS studies using microarray technology have played a fundamental role in the characterization of new genetic variants associated with diseases and other complex human traits [1-3]. Recently, CNVs have been identified as potentially responsible for a significant proportion of human phenotypic variability that remains unexplained [4,5]. With this objective in mind, several studies have provided finer-scale details of this type of variation and have enabled accurate CNV mapping of the human genome to be conducted [6-10]. The results from most of these studies have been compiled in the DGV database [11], the reference database for CNV studies.

Current studies agree that CNV regions collectively span between 10% and 20% of the genome, although the majority of these loci correspond to rare events with very low population frequencies (<0.05%) [6]. However, if we compare any two individuals in the general population, those CNVs with frequencies = 1% will be responsible for 90% of the total copy number variation between them [12]. These relatively frequent CNVs are also called Copy Number Polymorphisms (CNPs). To date, more than 1,000 CNP loci have been identified and analyzed [12-14]. Genetic population differences could lead to the identification of new CNP loci [6,7,12]. Consequently, although the present CNP maps are useful as a reference, an exhaustive list of CNPs is necessary for analyzing new sample collections on different populations.

Length estimates of CNP loci have decreased from ~500 kb to ~50 kb recently. Approximately 95% of CNV loci larger than 100 kb correspond to rare CNVs with non-polymorphic frequencies, while the majority of CNP loci range from 10 kb to 100 kb [6,12,13]. Therefore, for exhaustive identification of CNPs associated with disease, probe density must be increased and the sensitivity of the analytical methods improved.

Several technologies such as multiplex ligation-dependent probe amplification and array comparative genomic hybridization can be used to characterize CNV genotypes. However, the majority of these technologies cannot be used on a genome-wide scale for CNP analysis owing to low throughput or elevated costs. SNP probe microarrays have been proven to be highly robust for the characterization of the genotypes of these markers. It has been shown that, despite having a relatively low signal-to-noise ratio, they can be useful for characterizing CNVs. This has led several commercial companies, including Illumina and Affymetrix, to include non-polymorphic microarray probes for research. Therefore, it is a major challenge to develop analytical methods that can best deal with these deficiencies, avoid assignment errors, reliably quantify known CNPs and detect new CNV regions that could be associated with disease risk.

So far, most algorithms [15-18] covering CNV analysis have been based on the differences in the log R ratio (logR) and B-Allele frequency (BAF) measurements [19] between samples and a model learned from a reference set. One of the most frequently used methods for analysing CNVs using Illumina microarrays is PennCNV [6,18,20]. PennCNV is a CNV estimation method based on Hidden-Markov-Models (HMM), in which CNV calls are performed at the individual level by analyzing the sample logR and BAF values. This type of approach works well for large deletions and amplifications but it is very sensitive to intensity noise, leading to high false discovery rates [6]. New approaches have been developed based on the power of Gaussian Mixture Models (GMM) for modelling the statistical distribution of genotyping intensity data. The SCIMM method [16] uses GMMs over sets of informative probes but it has disadvantages including the inability to detect amplifications and the lack of public availability to the software. Another powerful approach is the Canary algorithm [21] but it was specifically designed for Affymetrix microarray data. Both the SCIMM and Canary methods, whilst they robustly genotype CNP loci, require a predefined set of CNP loci and cannot be applied to other available microarray probes.

In this study we present CNstream, a method for accurate whole-genome CNP identification and genotyping using Illumina Beadchip arrays. The algorithm is based on a robust single locus scoring algorithm followed by a segment-based genotyping algorithm - multilocus calling - that increases the sensitivity and the accuracy of the results. This method takes advantage of multiple sample analysis by jointly calling each probe and it increases the accuracy of the CNP calls by considering the scores of nearby and consecutive markers. The CNstream method is publicly available, fully implemented in R statistical software [22], and requires little user input, employing the direct output from Illumina genotyping software.

## Methods

### GWAS samples and quality control procedures

To evaluate CNstream and to compare its performance with PennCNV software, a cohort of 572 individuals from a published GWAS in Rheumatoid Arthritis (RA) was used [2]. Informed consent was obtained from all individuals, in accordance with the Declaration of Helsinki. The cohort was divided into 379 patients diagnosed with RA according to the American College of Rheumatology Criteria [23] and 193 control subjects. Individuals were genotyped using the Illumina HumanHap 300 genotyping microarray according to the protocol [2]. Raw intensity data were loaded into the Genomestudio software (Illumina, USA), which automatically performs a normalization step using affine transformation [19] at the sub-bead pool level (i.e. sets of beads that are manufactured together and behave similarly). The normalized intensity data were extracted, as were the BAF and logR values.

In order to ensure the quality of the data for the different CNP analyses we applied a series of quality control (QC) steps. Those individuals in which the percentage of successfully genotyped SNPs was lower than 95% were discarded [24]. Samples with excessive signal noise, defined as a logR standard deviation greater than 0.25 or an absolute value of the logR mean greater than 0.1, were eliminated [6]. QC metrics were computed exclusively using the autosomal probes.

### PennCNV analysis

PennCNV version 1.05 [18] was used to perform the CNV calls. The algorithm implemented by this software uses a Hidden Markov Model (HMM) [25] to analyze each sample, where the hidden states correspond to the number of copies and the observations are the BAF/LogR measurements. Emission probabilities are modelled as distributions depending on the expected LogR values for each state and the minor allele frequency of each probe. The transition matrix between consecutive markers also depends on the spacing between these markers.

The PennCNV output form includes all the sample calls, specifying the sample for each call, the chromosome region, the number of markers affected by the call and the copy number state. To avoid false positives and excessively large calls, the resulting CNV calls were filtered to focus analysis on copy number variants shorter than 1 Mb. Therefore, those calls greater than 1 Mb or including more than 100 probes were excluded, as they usually correspond to very rare CNVs or to PennCNV calling artefacts. In addition, samples with more than 100 CNV calls or with CNV calls spanning more than 7 Mb were excluded as this normally indicates poor DNA quality [26]. These quality control filters were based on previous CNV studies [6,26], and we verified that after observing the distribution of the inclusion thresholds of the parameter, the discarded samples were clear outliers (data not shown).

After removing calls that did not pass the quality control filters, we used PLINK software [27] to perform a region-based frequency filtering, as we were only interested in CNVs with frequencies higher than 1%. Consequently, PLINK software removed all CNV calls that completely span regions with four CNV calls or fewer. CNP regions were defined in the remaining calls as segments of consecutive probes spaced less than 100 kb. DGV matching was considered as an overlap between these CNV regions and the DGV regions. In order to test for CNV association, we used PLINK to run a simple permutation-based test of association of segmental CNV data for case-control phenotypes, performing 50,000 null permutations to generate empirical *P*-values. These *P-*values were calculated as (R+1)/(N+1), where R is the number of times the association statistic calculated from the permuted data (logistic regression coefficient in this case) was greater than the observed statistic, and N is the total number of permutations. In the present analysis, events (deletions or amplifications) had to be more frequent in the experimental group than controls and the test was performed for all events within a 50 kb slicing window.

### CNstream software

**Figure 1:**
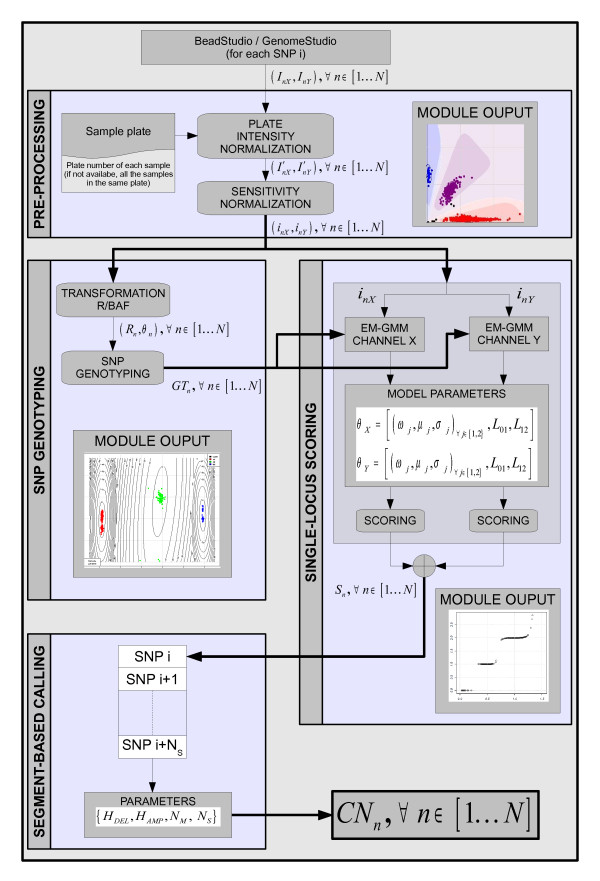
**Flowchart of CNstream processing steps**. Data processing with CNstream is organized into four functional modules: Pre-processing, SNP genotyping, Single-locus scoring and Segment-based calling.

CNstream is an R-statistical software package for whole-genome CNV discovery and genotyping specifically adapted for Illumina arrays. Consequently, the required data for the analysis can be directly extracted from GenomeStudio without a formatting step. The X and Y channel intensities (measuring A and B alleles, respectively) for each sample *n *are mandatory (*I*_*nx*_, *I*_*ny*_). The following subsections describe each of the CNstream processing steps (Figure 1).

#### First step: Pre-processing

**Figure 2:**
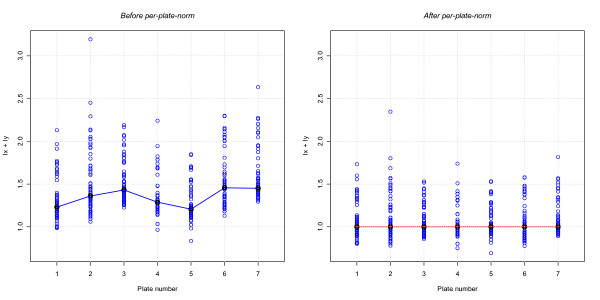
**Per-plate intensity normalization**. An example of how per-plate normalization can improve data quality. After this normalization step the plate intensity distributions follow a more similar pattern.

Intensity normalization is a very important step in microarray-based analyses as it ensures the accuracy of results. Therefore, although the data were normalized by Illumina genotyping software, per-probe intensity distributions of samples processed in different plates can demonstrate significant deviations. Generally, large studies involving hundreds or thousands of samples are processed using 96 well plates. Samples in this type of plate normally share similar environmental conditions throughout the labelling and hybridization processes and similar scanning parameters. These conditions could result in variability between samples in different plates and in the same plate. In order to reduce inter-plate variability, CNstream users can include the plate number of each sample, and a subsequent normalization step can be applied to standardize the plate intensity distributions over each marker (Figure 2). Then, for each plate *p *∈ 1K *P*,(1)

where N and P respectively denote the total number of samples and the total number of plates, *I*_*nc *_refers to the intensity of the sample n in channel C, and  is the indicator function for sample n genotyped in plate p. To improve the performance of this approach, a minimum threshold of five samples per plate was estimated.

After per-plate normalization, a scaling correction step is applied in order to equalize the overall sensitivity of both channels X and Y [19]. Medians are computed for the channel X intensities of candidate AA homozygotes to define an x-axis correction parameter *T*_*x*_, and for the channel Y intensities of candidate BB homozygotes to define a y-axis correction parameter *T*_*y*_:(2)

#### Second step: SNP Genotyping

Once the normalized intensities (*i*_*nX*_, *i*_*ny*_) have been computed, a clustering algorithm is applied to determine the SNP genotype (i.e. allele A homozygotes, allele B homozygotes and heterozygotes) of each sample. The approach used was allied to that described by Teo et al. [28]. It is important to note that for specific CNV probes, all samples will be genotyped as homozygote probes as only one allele will hybridize the DNA.

Using the normalized intensities, we compute the absolute normalized intensities *R*_*n *_= *i*_*nX *_+ *i*_*ny *_and the B-allele frequencies θ_*n *_= (2/π) arctan (*i*_*nX*_/*i*_*ny*_). The histogram of θ_*n *_values is used to determine the genotype centres and to cluster the samples following a minimum-centre-distance criterion. The mean and the variance of the samples assigned to each genotype are used to initialize the Expectation-Maximization (EM) algorithm [29] with a two dimensional (*R*_*n*_, θ_*n*_) Gaussian Mixture Model (GMM) with three components (allele A homozygotes, allele B homozygotes and heterozygotes). After one EM round, the final GMM parameters are accurately obtained (excluding very rare SNPs (<<1%) with poor clustering performance) and the genotypes of each sample are assigned following a maximum-likelihood approach.

#### Third step: Single-locus scoring

**Figure 3:**
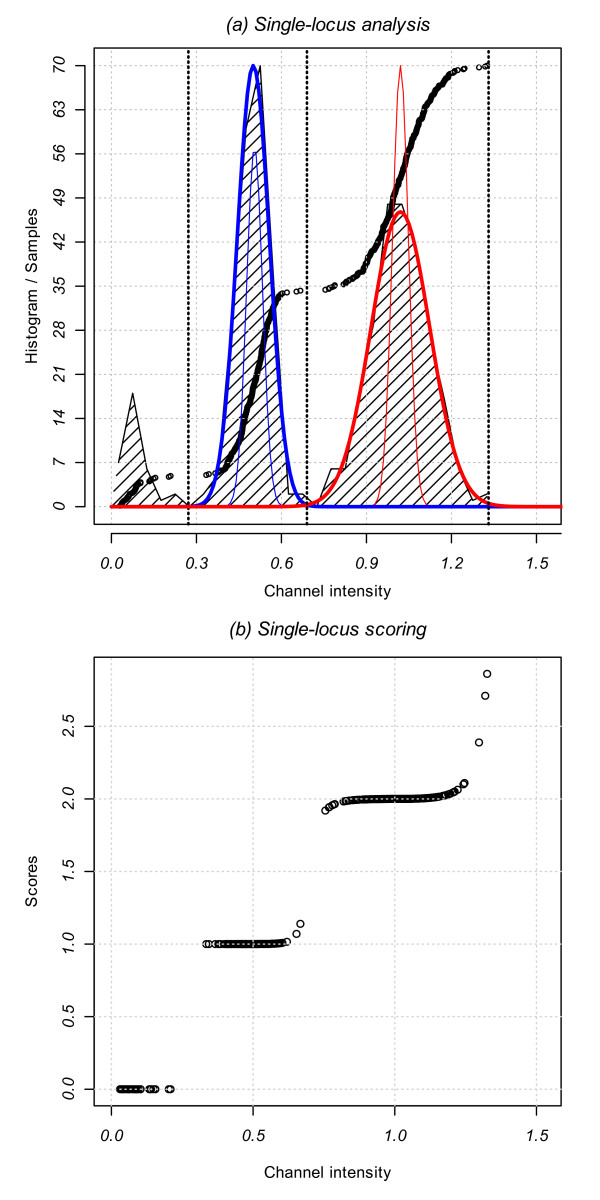
**Single-locus analysis**. (a) CNstream performs a GMM fitting over the intensities of the samples on each channel (black circles). The zero copy intensity thresholds are determined and a two-component GMM is fitted (one copy in blue and two copies in red). The thin colour lines represent the initial model, and the thick lines represent the final model after EM fitting. (b) Channel intensity of the samples for one channel within one probe versus scores assigned by CNstream.

Copy number scores for a probe *i *are computed by combining the estimated number of copies of each intensity channel for each sample, *n*. The single-locus approach separately analyzes each channel by combining the intensity distribution information and the estimated genotype cluster of the sample (Figure 3). We consider the analysis of channel X intensities, measuring the number of copies of allele A. The analysis of channel Y intensities is carried out in the same manner but it is reciprocal as it measures the number of copies of allele B.

In the third step, CNstream computes the zero intensity threshold (*L*_01_) so that all samples with lower intensities have a 0-score for this allele (i.e. 0 copies of allele A). This threshold is calculated by creating a vector with the sorted sample intensities delimited by two predefined limits and localizing the maximum increase of intensity between two consecutive samples in this vector. In the most common case, the lower intensity limit is defined by the intensity mean of the allele B homozygote samples as they have 0 copies of the allele A. The higher intensity limit is defined by the intensity mean of the heterozygote samples as the majority will have one copy of allele A. Once *L*_01 _has been computed, those samples with lower intensities are excluded from further analysis.

Having identified the zero thresholds, a GMM with two components is fitted to the intensity data of the remaining (non-zero) samples via the EM algorithm. The two GMM components will model the intensity distributions of the one-copy (A) and the two-copy (AA) samples, respectively. In order to improve the EM convergence time and the model fitting, the two GMM components were initialized using the SNP genotype information computed in the previous step (θ_0_): 1-copy component with the mean and the variance of the intensities corresponding to heterozygotes, and the 2-copy component using the allele A homozygote intensities:(3)

where  is the indicator function for sample n being a heterozygote if X = 1, or being a homozygote in the allele that is being measured (in this case AA) if X = 2. The EM algorithm was stopped after 15 iterations or if the increase in the Log-Likelihood Ratio between consecutive iterations was <0.1. The former condition is rarely reached and the EM converges in >99% of cases.

Once EM has fitted the model to the data, CNstream uses the maximum likelihood parameters obtained(4)

to assign a score  to each sample n at the analyzed probe *i *(Figure 3). CNstream also computes the intersection point between the two GMM components (*L*_12_):(5)

Scores ranging from 0 to 2 are assigned depending on the interval that holds the sample n intensity *i*_*nX*_:(6)

where .

At this point, samples that can hold more than two A allele copies are detected. In order to identify such samples, scores ranging from 2 to 3 are assigned to samples having intensities higher than μ_*F*2_. The maximum score, 3, is achieved when the sample intensity is more than three standard deviations σ_*F*2 _away from μ_*F*2_:

The value of three standard deviations was determined empirically by averaging the intensity distances of those samples that have amplifications. Once the probe scores of channel X and channel Y were computed, the final score for each sample *n *was calculated as follows:(7)

#### Fourth step: CNstream: segment-based calling

After single-locus scoring, CNstream analyzes the scores obtained for each sample along a set of consecutive and nearby probes, referred to as segments. Segments are defined by sets of *N*_*S *_consecutive probes (sorted by chromosome and base-pair), where the distance between the most distant probes does not exceed the maximum distance *D*_max_. For each probe segment [*i*, *i *+ 1K *i *+ *N*_*S*_-1], the scores  for each sample *n *are used to call the copy number of the sample on this segment. This calling is based on the definition of a deletion threshold *H*_*DEL *_and an amplification threshold *H*_*AMP*_, and on assigning amplifications and deletions to those samples that have a sufficient number of scores *N*_*M *_over the segment exceeding these thresholds (Additional file 1, Figure S1):(8)

where  refers to the copy number state assigned to the sample *n *within the probe segment [*i*, *i*+ 1... *i *+ *N*_*s*_-1].

### CNstream analysis

All samples that did not pass the quality control procedures were excluded from the CNstream analysis. Samples identified as outliers in the PennCNV calls (i.e. those with more than 100 CNV calls or with CNV calls spanning more than 7 Mb) were also excluded.

Once the computations were performed, the output of CNstream included all those segments where the number of amplification/deletion calls exceeded the minimum frequency threshold for a CNP (~1%). This segment-based analysis was performed using parameters that have been shown to obtain the best compromise between called CNP regions and correlates with the DGV database. *N*_*S *_= 5 was the optimal parameter value, which is in agreement with previous studies [30]. A minimum of *N*_*M *_= 3 scores below the deletion threshold *H*_*DEL *_to call a deletion, or 3 scores higher than the amplification threshold *H*_*AMP *_to call an amplification. The maximum distance *D*_max _allowed between markers in one segment was set at 100 kb. In order to calculate the frequencies at each CNP locus, a CNP region was defined as all the overlapping *N*_*S *_= 5 segments showing a common signal. Based on these counts, CNstream can also perform an association analysis using Fisher's exact test, provided that a file with the Case/Control status is included. In this case, the *P*-value, Odds Ratio (OR) and frequencies were included in the output.

### Selection of CNP candidate loci

From each of the two CNP analyses, loci showing evidence of association with RA susceptibility were selected (Table 1). Therefore chromosome 8 locus chr8:145,079,175-145,090,342 within the transcribed region of the Plectin 1 (*PLEC1) *gene was selected for PennCNV. Chromosome 8 locus chr8:15,435,527-15,467,031, within the transcribed region of the Tumour Suppressor Candidate 3 (*TUSC3*) gene was selected for CNstream. Both CNPs are on chromosome 8 but they are more than 100 Mb apart, making them two independent candidates.

**Table 1:** Significantly associated regions detected by CNstream and PennCNV

REGIONS	CHROM	BASEPAIR	DELS CASES		DELS CONTROLS		OR
				
			N	%	n	%	
CNSTREAM SIGNIFICANT SEGMENTS							

**8p22**	**8**	**15435527..15453141**	**24**	**7.2**	**4**	**2.6**	**2.9**
	
	**8**	**15439515..15455979**	**33**	**9.9**	**5**	**3.3**	**3.2**
	
	**8**	**15447669..15464497**	**33**	**9.9**	**5**	**3.3**	**3.2**
	
	**8**	**15450330..15467035**	**24**	**7.2**	**4**	**2.6**	**2.9**

19p12	19	20368239..20449621	51	15.2	11	7.2	2.3
	
	19	20385941..20473895	54	16.1	11	7.2	2.5
	
	19	20423788..20520617	54	16.1	11	7.2	2.5
	
	19	20439390..20522325	40	11.9	5	3.3	4

PENNCNV SIGNIFICANT PROBES							

**8q24.3**	**8**	**145079175**	**30**	**9.0**	**0**	**0.0**	**Inf**
	
	**8**	**145083192**	**30**	**9.0**	**0**	**0.0**	**Inf**
	
	**8**	**145083193**	**29**	**8.7**	**0**	**0.0**	**Inf**
	
	**8**	**145090342**	**29**	**8.7**	**0**	**0.0**	**Inf**

11p15.5	11	1073364	16	4.8	2	1.3	3.8
	
	11	1074362	16	4.8	2	1.3	3.8
	
	11	1074363	15	4.5	2	1.3	3.5
	
	11	1086494	15	4.5	2	1.3	3.5

19p13.3	19	4020119	15	4.5	1	0.7	7.1
	
	19	4028096	15	4.5	1	0.7	7.1
	
	19	4037807	15	4.5	1	0.7	7.1
	
	19	4041113	15	4.5	1	0.7	7.1

Number and percentage of cases and controls in each of the associated CNP regions detected by CNstream and PennCNV. In bold, the candidate regions chosen for quantitative PCR validation.

### Quantitative PCR validation of CNP loci

To determine the relative copy number at the two candidate loci we used Taqman Real-Time PCR technology. Recently, a panel of more than 1.6e6 pre-designed CNV Taqman assays has become available (Applied Biosystems, CA, USA), allowing high density coverage of almost all CNVs in the genome. Using this panel, the RT-PCR assay within the identified CNP locus and closest to the Illumina probe showing the strongest evidence of copy number variation was selected. Therefore, in PennCNV analysis, the probe with the highest degree of overlapping CN regions was selected; for CNstream analysis the probe with the highest number of CN-harbouring individuals was selected. For PennCNV validation, the Taqman assay "Hs00837735_cn" (closest to rs11136336 SNP probe, bp 145,079,175) and within the CN region described in DGV (chr8:145,064,090-145,740,218) was used. For CNstream RT-PCR, the "Hs03676276_cn" assay was utilised as it was in close proximity to the rs1346590 SNP probe (bp 15,453,141 of chromosome 8), and within the same DGV region (chr8:14,670,571-15,809,077). In order to include a 2-copy endogenous control within each RT-PCR reaction, the *RNAse P *Copy Number Reference Assay (Applied Biosystems) was utilised.

In this technical validation analysis a total of 30 individuals were genotyped: 10 having a deletion in the *TUSC3 *locus as defined by PennCNV, 10 having a deletion in *PLEC1 *as defined by CNstream and 10 without a CN in either of the two loci. In addition, individuals identified as carrying one CNV by one method were considered negative for the other method and therefore expanded the negative control group to 20 individuals in each case. For each assay, 5 ng of genomic DNA was assayed in triplicate in 10 μl reactions containing 1× final concentration TaqMan Universal Master Mix (Applied Biosystems, part number 4304437). Cycling was performed under default conditions in 384-well optical plates on an ABI 7900HT machine. Copy numbers for each sample and for each locus were inferred using the ΔΔCt method. In this method, the threshold cycle values (Ct) for each target Copy Number (TUSC3 and PLEC1) are normalized against the RNAseP cycle value (ΔCt = C_T, TARGET_- C_T, RNAseP_), which is known to be diploid. The resulting ΔCt values of each sample were compared with the average values of a control group (ΔΔCt = ΔC_t_-ΔC_t, control_) whose samples are known to be diploid over the targeted Copy Number regions. The estimated Copy Number for each sample was computed as 2·2^-ΔΔCt^.

### CNV analysis of Hapmap reference samples

Further evidence relating to the sensitivity of CNstream was provided using available data from Hapmap reference samples. In particular, the set of eight Hapmap samples thoroughly characterized in the study of Kidd et al. [8] were utilised. The list of CNVs from these individuals was downloaded from the DGV database (Additional file 1, Table S3).

Maximal coverage concerning genomic variability was obtained by downloading data from the Illumina Human1-Duo array, which characterizes 1,119,187 markers per sample. Raw intensity data from these individuals were downloaded from the Gene Expression Omnibus database (series GSE16896, GSE16895 and GSE16894). Given that CNstream is a sample-based algorithm, the complete set of 269 Hapmap individuals was used to perform CNV calling. PennCNV estimates CNVs at the individual level, allowing the analysis to be limited to the eight specific Hapmap samples. In addition, a segmentation-based method was also utilised, which is a different approach from GMM or HMM-based methods. The Circular Binary Segmentation algorithm (CBS) described by Venkatraman et al. [31] was used. Like PennCNV, this method estimates CNVs at the individual level, and is available as an open-source R-package from the Bioconductor repository [32].

## Results

### Quality control analysis

From the samples described in the methods section, 53 individuals were excluded owing to a high standard deviation over their logR values, seven samples were excluded because the mean logR values were over the threshold and 10 were excluded for fulfilling both conditions. Of the remaining 502 samples, five were excluded as they had an excess of PennCNV calls. CNstream was carried out using the plate normalization option and nine samples were excluded from plates with insufficient sample to estimate this parameter accurately. The final cohort analyzed using PennCNV and CNstream consisted of 488 samples, 335 of which were from RA patients and 153 from controls.

### PennCNV analysis results

After running PennCNV over the 488 samples that passed the QC filters, the number of CNV calls was 7,321, with a mean of 15 calls per individual. PennCNV delivers a report file with relevant fields for each call such as chromosomal region, number of probes per CNV, the estimated copy number and the sample name. As this study concerned CNP regions (i.e. CNVs with frequency higher than 1%), the PLINK frequency filter was applied to remove probes having a CNV frequency lower than 1%. This resulted in a final set of 2,050 probes with a positive signal (Additional file 1, Figure S2a). These positive probes were joined by physical proximity to obtain a final set of 283 CNP regions. The correlation of these regions with the DGV database was high; 232 regions (82%) had overlapping CNV regions previously defined in the DGV database. The majority of these 283 regions (62%) had lengths ranging from 10 Kb to 100 Kb [6,12], with a mean length of 70 Kb (Additional file 1, Figure S2c). The majority of these regions had a CNV frequency ranging from 1% to 3%, with only 63 regions exceeding 3% frequency (Additional file 1, Figure S2b).

PLINK was used to run a simple permutation-based test of association of segmental CNV data for case/control phenotypes, performing 50,000 null permutations to generate empirical *P*-values. Three hundred and forty-six probes presented with an empirical p-value lower than 0.05 (Additional file 1, Figure S2d) and 89 were found within three genomic regions showing the highest significance values (Table 1). The most associated region corresponds to a high frequency 8q24.3 deletion, containing 50 probes with empirical *P*-values of association lower than 0.01. Within this region, the segment ranging from base pair 145,079,175 to 145,133,612, within the *PLEC1 *gene coding region, yielded the highest global significance values; 14 probes had an empirical *P*-value below P = 7e-5. This segment has been previously defined as a CNP region in several studies [6,7,18,33]. As this region demonstrated the strongest association to RA susceptibility, it was chosen for posterior CNV validation using Taqman Real-Time PCR technology.

The second significant region corresponded to a 11p15.5 deletion locus (24 probes). Within this region, the segment ranging from base pair 1,028,110 to 1,074,363, within the mucin 2 (*MUC2*) transcribed region had 10 probes with empirical *P*-values lower than 0.005. This segment has been defined as a CNP region by several studies [7,9,10]. The last significant region identified by PennCNV analysis corresponds to a 19p13.3 CNV locus within myeloid/lymphoid or mixed-lineage leukaemia gene (*MLLT3*); 15 probes passed the significance threshold, with nine having empirical *P*-values lower than 0.005.

### CNstream results

To compare the performance of CNstream with a well-established method such as PennCNV, the CNP analysis was carried out using the same sample set that passed all the QC filters (n = 488 samples). The report generated by CNstream included relevant information concerning those probe segments that exceeded the minimum frequency threshold including the chromosome position, the percentage of amplifications or deletions, the *P*-value (if the phenotype file was provided) and the CNV call for each sample.

**Figure 4:**
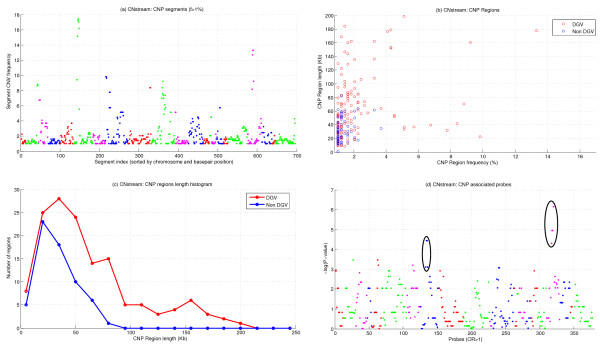
**CNstream results**. (a) CNV frequencies of the 697 segments of five consecutive probes that exceeded the 1% frequency filter, sorted by chromosome and basepair position. (b) Length and frequency distribution of the 206 CNP regions. The colours indicate whether the regions match a DGV region or not. (c) Comparison between the CNP length histograms of the CNP regions. (d) *P*-values of the 353 segments with an OR > 1. Associated regions in 8p22 and 19p12 have been highlighted.

After running CNstream, 697 segments were obtained and more than 1% of the samples had a CNV (Figure 4a). Subsequently, overlapping segments were merged to obtain a final set of 206 regions, of which 143 (69.4%) matched previously defined DGV regions. Regarding the length and the frequency distribution of these 206 regions, 85% of these CNVs had lengths ranging from 10 Kb to 100 Kb, with only 24 regions exceeding the 100 Kb limit, although all matched to DGV regions (Figure 4c). CNP frequencies were predominantly between 1% and 3%, with 24 regions exceeding the 3% threshold (Figure 4b). For each segment, the CNstream output included the OR and the resulting *P*-value after performing a chi-square association test. Excluding segments with an OR lower than one (i.e. regions where the CNV is more common in patients than in control subjects), a final set of 377 segments was obtained, from which two regions stand out according to their significance values.

The most significant CNP locus was a 8p22 deletion between bp 15,439,515 and bp 15,464,497, which had a 9.9% frequency in the experimental group compared with a 3.3% frequency in controls (*P-*value = 0.019). This region was obtained after merging four consecutive 5-probe segments (Table 1) with OR values higher than 2.9 and a maximum OR of 3.2 in the central segments (i.e. 33 RA patients carrying a deletion vs. five controls carrying the same deletion). This 8p22 CNP is located in the TUSC3 transcribed region, and matches previously defined DGV regions [6,8,9,12,13,18,33,34]. This CNP had the highest evidence of statistical association to RA after CNstream analysis, therefore Taqman Real-Time PCR validation was performed in this region.

**Figure 5:**
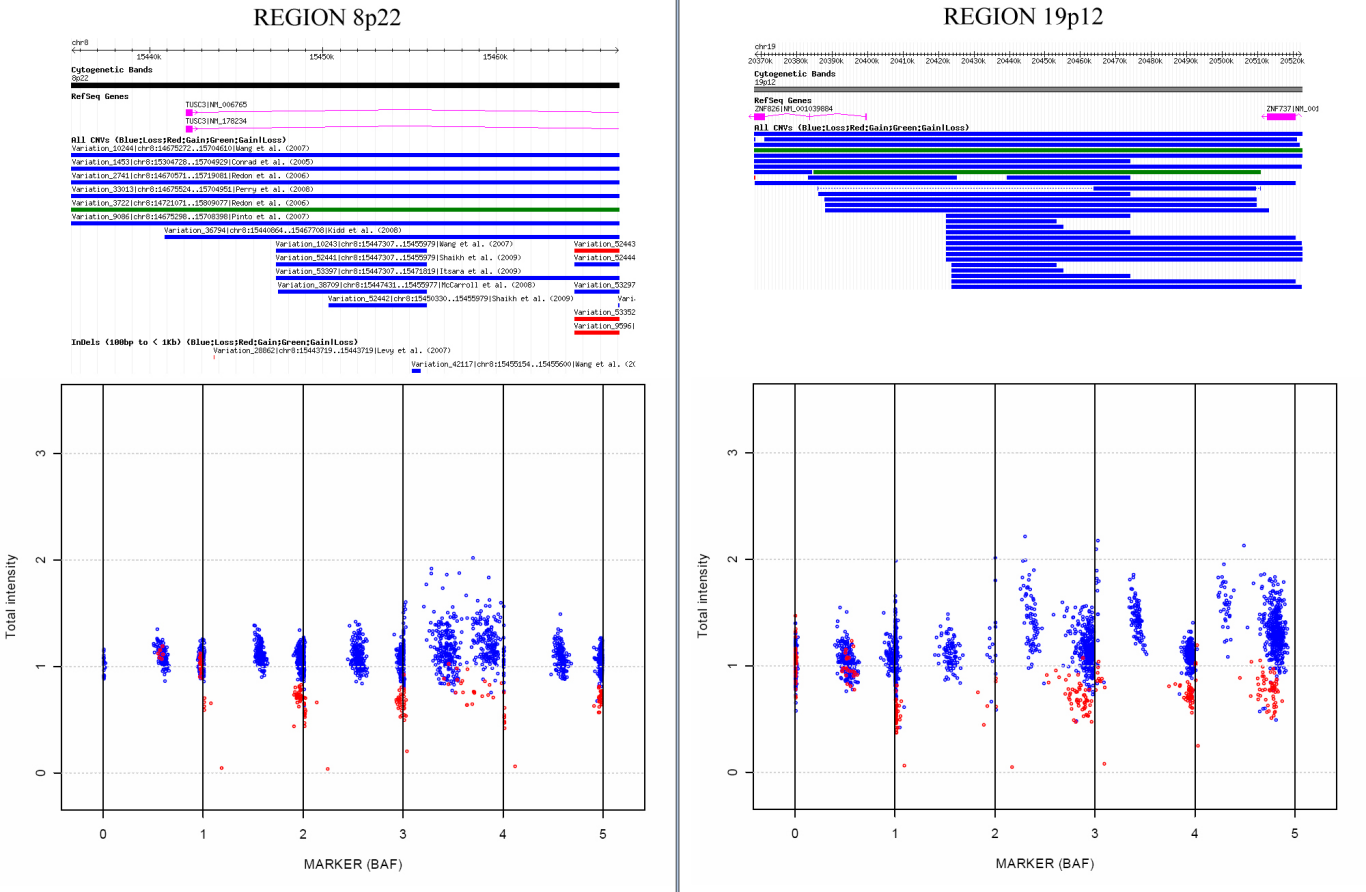
**CNstream: Top candidate regions**. The top figures present the information detailed in the DGV for the two most associated regions detected by CNstream. Both have been previously identified as CNV regions and, principally, as deletion loci (blue lines). The lower figures demonstrate the distribution of the BAF and the normalized intensities along the five probes of the segment for the samples that have deletions (in red) and those that are detected as diploid (in blue).

A second associated region was also detected in 19p12, where deletions between base pair 20,368,239 and base pair 20,439,390 were found to be more common in the experimental group than in controls (16.1% versus 7.2%). This region is located in the Zinc Finger Protein 826 (*ZNF86*) transcribed region and matched previously defined DGV regions [6,8,9,12,16,18,33]. Detailed information regarding these regions is presented in Table 1 and Figure 5.

### Quantitative RT-PCR validation of CNP regions

**Figure 6:**
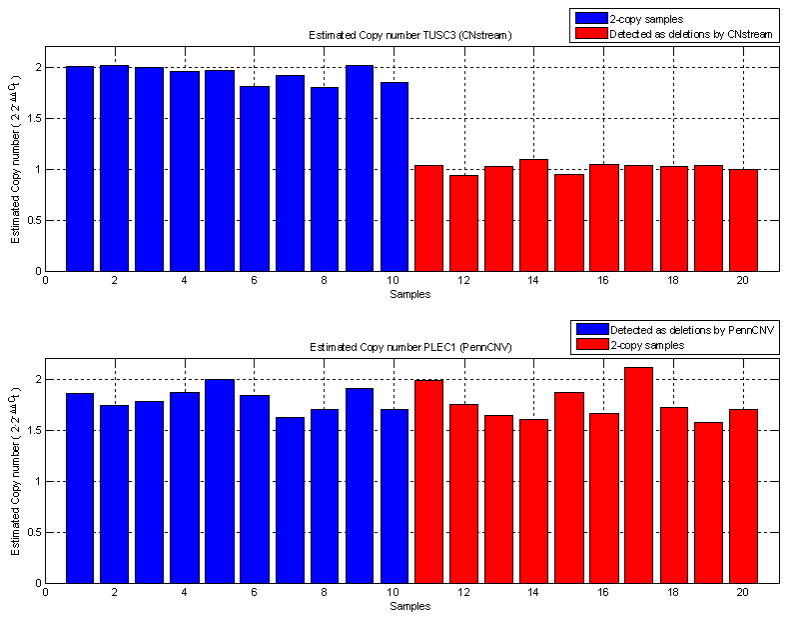
**Quantitative PCR results**. (a) ΔΔ*C*_*T *_values for 20 individuals for the *TUSC3 *locus, which clearly validate the deletions assigned by CNstream (red bars) versus the 2-copy samples (blue bars). (b) ΔΔCt values for the 20 individuals analyzed for the *PLEC1 *locus demonstrate no differences between those that were called with a deletion (in blue) and those that were called as 2-copy samples (in red) by PennCNV.

Taqman Real-Time PCR technology allowed the results of the most important candidate locus for each algorithm to be compared: the 8p22 deletion locus (*TUSC3*) for CNstream and the 8q24.3 deletion locus (*PLEC1*) for PennCNV. For the CNstream candidate the CNV calls were replicated, with all deletion-carrying individuals having ΔΔCt values close to one (CN = 1) and diploid individuals having a ΔΔCt close to zero (CN = 2) (t-test *P-*value = 4.82E-14, Figure 6). For the PennCNV candidate, however, ΔΔCt values for all individuals were close to zero (CN = 2) with no significant difference between deletion-carrying individuals and the 2-copy samples (t-test *P-*value = 0.54, Figure 6).

### Comparison between PennCNV and CNstream results

**Figure 7:**
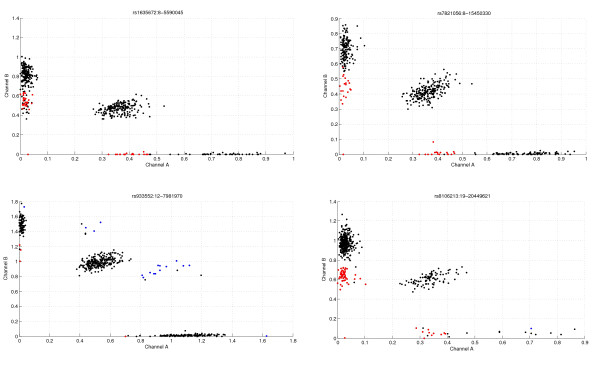
**Cnstream: Sensitivity gain**. Intensity plots of several probes of those loci where CNstream made more calls than PennCNV (Table 2). Samples called as deletions are plotted in red and those with amplifications in blue. As shown, the intensity measurements of these samples match a typical CNV pattern. However, several loci match the CNV pattern but are assigned to the normal state. This is because these particular samples do not show consistent behaviour throughout the adjacent probes that are analyzed by CNstream.

The CNP regions detected by CNstream and PennCNV were compared. Both sets of CNP regions were matched and 103 regions were detected by both methods. These overlapping regions were used to compare the number of CNV calls identified by both algorithms. In 49 of the 103 regions, the difference between the numbers of calls assigned was, at most, 1. Analysis of the other 54 CNP regions revealed that CNstream had an increased call rate in 28 of them and PennCNV had an increased call rate in the remaining 26. In the CNP regions where the number of calls was most discordant (Table 2), it was manually verified that for CNstream analysis the calls had a clear CNV pattern, supporting the existence of an underlying CNP (Figure 7).

**Table 2:** Comparison in the number of calls between CNstream and PennCNV

REGION	CNstream	PennCNV	REGION	CNstream	PennCNV
chr1:2151995..2236917	11	5	chr2:242633058..242645262	5	13

chr3:65166887..65187636	33	22	chr3:75511365..7553579	7	41

chr6:95118323..95118537	10	5	chr5:32142841..32171356	6	11

chr8:5590045..5591685	48	25	chr8:145064091..145083192	6	23

chr8:15447669..15455979	38	23	chr10:47013328..47173619	45	65

chr10:90934639..90954006	25	12	chr10:135116379..135167074	5	24

chr12:7884583..8017012	21	5	chr10:13522971..135284293	10	24

chr14:85518391..85557882	22	5	chr17:74878104..74905197	9	21

chr16:1192442..1265972	13	7	chr19:48267714..48350666	6	16

chr16:1744358..1781034	28	13	chr19:59423491..59445355	7	34

chr19:681297..717103	11	6	chr19:59994795..60018551	21	38

chr19:20385941..20528316	65	7	chr20:14815422..14856741	5	13

chr20:61323074..61366354	12	5	chr20:35443071..3548526	5	21

chr21:45756756..45788806	10	5	chr21:18981497..19000295	5	12

			chr21:43647907..43659936	6	12

			chr22:21169096..21190977	6	12

			chr22:21223788..21337263	6	12

			chr22:2408234..24235221	21	27

Comparison of the number of CNV calls made by both algorithms in the CNP regions that were detected by both algorithms and that showed the maximum difference in number of calls.

### Comparison of CNV detection on Hapmap reference samples

**Figure 8:**
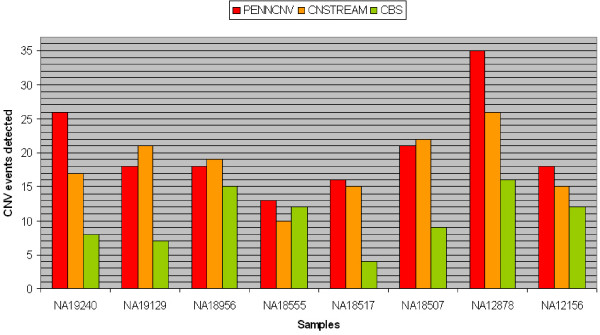
**Quantitative PCR results**. Number of CNV events detected by CNstream, PennCNV and CBS over the eight Hapmap reference samples characterized in Kidd et al.

A similar trend was found in the Illumina genotype data and a reference Hapmap dataset (Figure 8). Comparing the results of CNstream with PennCNV, a similar number of calls was achieved over the CNVs validated by Kidd et al. PennCNV matched 165 CNV loci over the eight samples (6.98%) while CNstream matched a total of 145 CNV loci (6.13%), indicating that both algorithms have similar sensitivity over this particular dataset. Within these calls, 92 CNVs were detected by both algorithms, demonstrating that CNstream can increase the sensitivity by >30%.

CBS, the segmentation-based method, was not as accurate as the other two methods (Table 3). Furthermore, CBS results needed to be curated as a number of CNV calls were clear artefacts that spanned regions of > 5 Mb (data not shown).

**Table 3:** Comparison in the number of calls between CNstream and PennCNV

SAMPLE	CNV events **from Kidd et al**.	PENNCNV	CNSTREAM	CBS	Events detected by Cnstream and PennCNV
NA19240	405	26	17	8	16

NA19129	339	18	21	7	15

NA18956	307	18	19	15	10

NA18555	383	13	10	12	4

NA18517	100	16	15	4	8

NA18507	174	21	22	9	14

NA12878	351	35	26	16	15

NA12156	305	18	15	12	10

TOTAL	2364	165	145	83	92

Number of CNV events detected by each algorithm for each of the eight Hapmap reference samples. Number of events detected simultaneously by CNstream and PennCNV is provided in the last column.

## Discussion

These results highlight the benefits of using CNstream, a method for whole-genome CNV discovery and genotyping for Illumina BeadChip arrays. The present method combined a single-locus scoring approach that takes advantage of the joint clustering analysis of all the intensity samples at each probe with the computational speed and analytical accuracy of estimating CNPs from segments of consecutive probes. Compared with PennCNV, CNstream has superior sensitivity in several common CNPs and it can identify different CNPs that are not detected by PennCNV. The CNP detected by CNstream was demonstrated to be the most significantly associated with RA susceptibility using available RT-PCR technology.

With regard to the single-locus scoring method, CNstream reduced the processing time by robustly initializing the GMM before the EM procedure. This allows a whole-genome analysis to be carried out without the need for previously defined CNP maps, which are required for the SCIMM method. In addition, in comparison with SCIMM, CNstream is able to detect amplifications. Furthermore, CNstream is available as a publicly available R statistical software package.

One of the main problems with single-locus CNV analysis is that some probes can have noisy intensities that could lead to erroneous CNV calls. For this reason, a stage of segment-based calling was added that took into account the CNV evidence over consecutive probes for each sample. This effectively minimized the effect of this type of error and increased the accuracy of the calls. An indirect measurement of the accuracy of the calls is the degree of overlap between the found CNP regions and those listed in the DGV database. The CNstream analysis of the present data demonstrated a 70% overlap with the DGV regions, indicating a strong correlation with previous studies. However, it is important to note that this does not mean that the remaining 30% CNP loci are false-positives. Manual inspection of the intensity plots revealed that these were likely to be true CNP loci. The fact that there are as yet no studies from Spanish cohorts included in the DGV database could explain why these regions are still not present in this public CNV resource. In the future, more specific studies with alternative technologies such as RT-PCR will be necessary to characterize this new group of CNP loci fully.

One of the most time-consuming aspects of genome-wide data analysis is formatting data files; CNstream requires minimal user input to perform a genome-wide CNP analysis with Illumina data. The input files can be easily extracted from Illumina genotyping software (i.e. Beadstudio or Genomestudio) and loaded into R for subsequent CNV analysis. There is a step-by-step tutorial to guide interested users: http://www.urr.cat/cnv/cnstream.html. The default parameters have been optimized for Illumina array data but experienced users can modify them using the available R function arguments. For example, users can seek to gain less or more specificity by varying the maximum segment length allowed (default set to 100 Kb), the number of markers per segment (default set to 5) or the score thresholds for amplifications and deletions.

Comparing the results obtained with CNstream with those obtained by PennCNV, we demonstrated that CNstream increases the total number of CNP regions detected by 36%. This is important as it demonstrates the consistency of the results between both analytical algorithms (i.e. 50% of the CNP regions detected by CNstream where also found using PennCNV). Is also worth noting the substantial increase in the number of different CNP regions identified (50% of the CNP regions detected by CNstream were not detected by PennCNV), which could be important risk variants for a particular disease or trait. CNstream had increased sensitivity in several of the 50% CNP regions called by both algorithms. This reduction of the false-negative rate could be critical in increasing the statistical power of genome-wide studies analyzing CNPs.

RT-PCR was used to validate the CNP most associated with RA susceptibility. CNstream CNP calls were validated but PennCNV CNP deletion calls could not be distinguished from diploid events in this region. The RT-PCR assays used have been validated to perform optimally with Taqman technology and all measurements were done in parallel. Therefore, it is unlikely that technical differences could explain the lack of replication of the PennCNV loci. Moreover, the regions targeted by these RT-PCR assays were the closest to the microarray probes that had high evidence of CNP presence by each of the CNV analysis methods. Therefore, more specific approaches such as targeted sequencing will be necessary to explain the lack of replication of the PennCNV CNP.

*TUSC3 *(also *N33*) is an 11 exon gene spanning approximately 224 kb that was first cloned from a homozygous deletion found in metastatic prostate carcinoma [35], suggesting a tumour suppressor role for this gene. It is under-expressed in ovarian carcinoma [36] although the presence of an underlying deletion that could explain the altered expression has not been investigated. *TUSC3 *is thought to be an ortholog of the yeast Ost3 protein, which is implicated in the transference of sugar residues to the nascent protein, and its expression is not restricted to a particular human tissue. In a recent study, a homozygous deletion in the *TUSC3 *locus was shown to be associated with a form of Autosomal Recessive Mental Retardation [37]. To date, no study has implicated *TUSC3 *deletion with RA or any other chronic inflammatory disease. In order to clarify the association of this gene with the genetic etiology of RA, it will be fundamental to replicate these findings in an independent dataset of RA patients and controls. Disposing of a robust RT-PCR assay like that used in the present study will facilitate the analytical process.

Another possible approach to comparing the performances of different CNV genotyping algorithms would be to use microarray data from well-characterized individuals such as the Hapmap samples. The CNV content of eight Hapmap samples validated in the study by Kidd et al. could be used as a reference dataset. In the present study, CNstream, PennCNV and CBS methods were used on this dataset to compare the sensitivities of each approach. CNstream and PennCNV analyses performed superiorly to the segmentation-based method on Illumina microarray data. CNstream is a sample-based method whereas PennCNV and CBS are individual-based methods; the sensitivity of the CNstream approach improved with an increased sample size. However, using a relatively small genome-wide dataset (n = 269), CNstream performed to a similar level to PennCNV. Therefore, in the sample sizes typical of GWAS studies of common traits, CNstream will be a valuable tool for researchers aiming to identify CNPs and perform genotyping.

## Conclusions

In the present study a new method, CNstream, for CNP identification and quantification using the Illumina microarray platform was developed. This genetic analysis tool was able to identify known CNVs and incorporate strong evidence in favour of new CNP loci. The increased sensitivity in several regions compared with PennCNV will increase the power of genome-wide scans for CNPs associated with diseases or other complex traits. Using CNstream, researchers performing GWAS analysis with Illumina platforms will be able to analyze the data for the presence of relevant CNVs associated with disease risk. We conclude that CNstream is a powerful and useful tool for CNP analysis of complex human traits.

## Competing interests

The authors declare that they have no competing interests.

## Authors' contributions

AA, AJ and SM conceived, designed and developed the CNstream method. RT and CC designed and performed qRT-PCR validation experiments. SM, JC, AB and JT acquired the study samples and the associated clinical data. AA, AJ and SM performed GWAS CNP analyses and wrote the manuscript. All authors have read, revised and approved the final manuscript.

## Supplementary Material

Additional file 1 **Supplementary figures and tables**. CNstream segment-based calling example, PennCNV analysis figures, and description of CNV events on well-characterized Hapmap reference samples. Click here for file
