# Supplementary material for: CNstream: A method for the identification and genotyping of copy number polymorphisms using Illumina microarrays
Source: BMC Bioinformatics. 2010 May 19;11:264. doi: 10.1186/1471-2105-11-264 (PMC3098064; doi:10.1186/1471-2105-11-264)
Supplement: Additional file 1 — Supplementary figures and tables. CNstream segment-based calling example, PennCNV analysis figures, and description of CNV events on well-characterized Hapmap reference samples. [file 1471-2105-11-264-S1.DOC]

**FIGURE S1. Segment-based calling**

CNstream jointly calls segments of N consecutive probes by analyzing the scores of each sample within these probes. This figure shows the scores obtained in 488 samples along 5 consecutive probes in a CNP-locus detected in chromosome 8. Those samples that have been finally assigned to a deletion are plotted in red (in this case, having at least three of the five scores under the deletion threshold). Segment-based calling avoids erroneous calls due to low-quality clustering probes (i.e. the fourth probe in this figure).

## FIGURE S2. PennCNV results

**(a) CNV frequencies of all the 2,050 probes that exceeded the 1% frequency filter, sorted by chromosome and basepair position. (b) Length and frequency distribution of the 283 CNP regions. The colours indicate whether the regions match a DGV region or not. (c) Comparison between the CNP length histograms of the CNP regions. (d) Significance values obtained by PLINK for the 346 probes with an empirical *P*-value lower than 0.05.**

| SAMPLE | POPULATION | CNV events | # AMPS | # DELS | Median  length | Median  markers |
| --- | --- | --- | --- | --- | --- | --- |
| NA12156 | CEPH | 305 | 142 | 163 | 26881 | 14 |
| NA12878 | CEPH | 351 | 168 | 183 | 24443 | 13 |
| NA18507 | YORUBA | 174 | 66 | 108 | 28438,5 | 14,5 |
| NA18517 | YORUBA | 100 | 25 | 75 | 32989 | 18,5 |
| NA18555 | CHINA | 383 | 162 | 221 | 26177 | 13 |
| NA18956 | JAPAN | 307 | 156 | 151 | 20986 | 10 |
| NA19129 | YORUBA | 339 | 164 | 175 | 25181 | 13 |
| NA19240 | YORUBA | 405 | 221 | 184 | 25403 | 14 |

## Table S3 - HAPMAP REFERENCE SAMPLES

This table shows the eight Hapmap reference samples characterized in Kidd et al.. The number and type of CNV events detected are provided as well as the median length and the median number of probes of the events.
